# S2-5 Physical Literacy in Austria: development of the Perceived Physical Literacy Questionnaire (PPLQ) for the adult population

**DOI:** 10.1093/eurpub/ckad133.013

**Published:** 2023-09-11

**Authors:** Peter Holler, Johannes Jaunig

**Affiliations:** FH JOANNEUM University of Applied Sciences, Institute of Health Management in Tourism, Bad Gleichenberg, Austria; University of Graz, Institute of Human Movement Science, Sport, and Health, Graz, Austria

## Abstract

**Purpose:**

Physical Literacy (PL) is a relatively new concept in Austria. However, PL research in Austria is currently centered around one research group and has focused exclusively on adults, a cohort which has been largely overlooked by the PL community so far. Recent projects included an intervention to increase PL in physically inactive adults and the development of the Perceived Physical Literacy Questionnaire (PPLQ). The PPLQ is a comprehensive but easy-to-use and easy-to-understand PL questionnaire for adults in German language. We describe the development process of this questionnaire in the following.

**Methods:**

The process subsumes several phases with the involvement of experts to ensure high content validity. In phase 1, a PL questionnaire was taken from our previous pilot studies for further improvement. This instrument was not yet validated, but already consisted in large part of established questionnaire (subscales) for six PL domains (motivation, confidence, physical competence, knowledge, understanding, and physical activity behavior). In phase 2, the revised 51-item instrument was tested in an online-survey (N = 506) and a shortened version was developed using exploratory factor analyses. The remaining 31 items were inspected using cognitive interviews (phase 3) and an external comprehensibility check to ensure language level A2 (phase 4). Subsequent results belong to phase 5, in which reliability as well as factorial and convergent validity were tested in another sample of 417 adults.

**Results:**

The assumed 31-item model revealed misfits on global and local level and was modified based on expert opinions and statistical considerations but remained with six PL dimensions. After reducing the complexity of the model, we found empirical support for a theory-compatible 24-item version. The global model-fit of the final model was acceptable with minor misfits at item level. Internal consistency (omega) and convergent validity results were satisfactory.

**Conclusion:**

The PPLQ is the first PL assessment instrument for a broad adult population that incorporates the holistic nature of the concept. However, further studies should attempt to improve the identified minor weaknesses and apply it in different language versions. These initial results are promising for the application in research as well as practice.

**Support/Funding Source:**

No specific funding.

